# Exosome-Associated circRNAs as Key Regulators of EMT in Cancer

**DOI:** 10.3390/cells11101716

**Published:** 2022-05-23

**Authors:** Laura Amicone, Alessandra Marchetti, Carla Cicchini

**Affiliations:** Department of Molecular Medicine, Sapienza University of Rome, Viale Regina Elena, 324, 00161 Rome, Italy; laura.amicone@uniroma1.it (L.A.); alessandra.marchetti@uniroma1.it (A.M.)

**Keywords:** extracellular vesicles, circular RNAs, epithelial-to-mesenchymal transition, tumor microenvironment (TME), epithelial tumor progression

## Abstract

Epithelial-to-mesenchymal transition (EMT) is a dynamic program of cell plasticity aberrantly reactivated in cancer. The crosstalk between tumor cells and the tumoral microenvironment (TME) has a pivotal importance for the induction of the EMT and the progression toward a malignant phenotype. Notably, exosomes are key mediators of this crosstalk as vehicles of specific molecular signals that include the class of circular RNAs (circRNAs). This review specifically focuses on the role of exosome-associated circRNAs as key regulators of EMT in cancer. The relevance of these molecules in regulating the intercellular communication in TME and tumor progression is highlighted. Moreover, the here-presented evidence indicates that exosome-associated circRNA modulation should be taken in account for cancer diagnostic and therapeutic approaches.

## 1. Introduction

Epithelial-to-mesenchymal transition (EMT) is a complex process by which epithelial polarized cells lose cell–cell interactions and acquire a mesenchymal migratory phenotype. Different EMT subtypes can be categorized according to when and where the process occurs: (i) type 1 concerns developmental and organogenesis events; (ii) type 2 plays a major role in wound healing, tissue regeneration, and fibrosis; and (iii) type 3 characterizes epithelial cancer. The reactivation of the transdifferentiation process, indeed, renders tumor cells able to metastasize, thanks to the acquisition of migration ability and invasiveness properties. The relevance of the EMT in cancer biology is further underlined by the evidence that EMT traits are often coupled to stemness properties, increased tumorigenicity, metabolic reprogramming, and a pro-survival phenotype in stress conditions. Therefore, therapeutic treatments targeting EMT could interfere with multiple aspects that foster tumor progression.

Several observations unveiled the dynamics of the EMT process that occurs and progresses throughout a series of gradual molecular and morphological changes of epithelial cell phenotype and that, in some cases, can revert through an inverse transdifferentiation process, named mesenchymal-to-epithelial transition (MET) [1,2,3,4]. Many of the intermediate states that the epithelial/mesenchymal cells acquire during these transitions can be more or less stably maintained thanks to a balance between several pro- and anti-EMT elements, so that many situations of partial EMT can be encountered. This is particularly relevant in cancer, where each intermediate state could show distinct properties of invasiveness and play specific roles in the malignancy.

At the base of the dynamism of these processes and of the variety of intermediate cellular phenotypes that can be observed, there are complex molecular mechanisms resulting from the cooperation of and the interplay between numerous regulatory and effector molecules, belonging to different pathways, many of which still have to be characterized. In this context, epigenetic mechanisms controlling the expression of the EMT/MET genetic programs are becoming increasingly relevant, especially those involving non-coding RNAs.

With the rapid development of sequencing technology, numerous circular RNAs (circRNAs), structurally distinct from other coding and non-coding RNAs, have been identified in human transcriptomes. Notably, the expression of circRNAs can be cell-type and cell-state specific, indicating their complex regulation and functional role in the cell [5]. Recent studies have revealed that circRNAs contribute to the growth and progression of several human cancers. Moreover, several studies proposed some circRNAs as prognostic biomarkers because their presence and level in body fluids can predict the final output of many pathological conditions [6,7,8]. Furthermore, the finding of circRNAs in biological fluids indicates that they may also be important mediators of communication between cells. These RNAs are turning out to be important biological regulators, acting as sponges for miRNAs, as scaffolds for proteins, and also as coding molecules.

In cancer, the crosstalk between tumor cells and other elements of the tumoral microenvironment (TME), as well as between tumor cells and distant tissues targets of metastasis, has a pivotal importance for the induction of the EMT and the transition toward a malignant phenotype [9]. This intercellular communication significantly impacts on the capacity of tumoral cells to acquire invasiveness and regulates EMT plasticity. In turn, it makes the tumoral and distant tissue microenvironments favorable to the tumor growth, the colonization of secondary sites and the metastasis formation.

In EMT and cancer, cell–cell communication is importantly mediated by extracellular vesicles (EVs), an heterogeneous class of lipid-bilayer structures, including exosomes and microvesicles, that differ for size and biogenesis and are virtually produced by all types of cells. EVs’ specific cargo (including proteins, lipids, DNA and RNAs) has a relevant functional impact on receiving cells and profoundly differs between untransformed and transformed cells, as well as between epithelial cells and their counterpart that has undergone EMT [10,11,12,13]. Notably, besides other regulatory non-coding RNAs, such as microRNAs and lncRNAs, circRNAs have been recently unveiled as important elements of the exosome and microvesicle cargo, where they can also be enriched with respect to the level present in producing cells. Moreover, recent studies specifically demonstrated the key role in cancer progression and in the EMT of circRNAs embedded in the exosomes derived from tumoral cells, as well as from cells of the tumor niche.

Therefore, this review focuses on the current knowledge about exosomal circRNAs with a role in the EMT, to provide readers with a state-of-the-art understanding of this specific research topic, still largely unexplored. The relevance of circRNAs in regulating tumor progression as well as the tumor cell-microenvironment network is highlighted. Moreover, the perspective section illustrates the potential therapeutic applications that the advances in the molecular functions of exosomal circRNAs promise.

## 2. Cell-to-Cell Communication in Tumor Microenvironment

A tumor is a complex and dynamic collection of transformed cells and different other cellular and non-cellular components that reciprocally interact. The microenvironment that surrounds tumor cells comprehends, indeed, both different cell types and several non-cellular elements. In particular, TME include fibroblasts, endothelial cells, adipocytes, immune cells, stem cells, and acellular components such as the extracellular matrix (ECM), EVs and a variable repertoire of cytokines, chemokines and growth factors. Moreover, physical and chemical elements, such as low pH and hypoxia, are critical microenvironmental elements. Tumor cells gradually build their own TME, conditioning its components to their advantage. Therefore TME, which at first can exert an antitumor effect, is progressively modified to become a fundamental element in promoting tumor growth and progression. The complex and dynamic interplay between cancer cells and TME cellular components, mediated by secreted factors and EVs, results in local inflammation, immunosuppression and angiogenesis and, eventually, in cancer cell survival, proliferation, local invasion, and dissemination, together with the acquisition of drug resistance. The EMT of tumor cells, a crucial element of malignancy, is a relevant outcome of the crosstalk between cancer cells and TME components [14].

Primary tumors and TME cells, moreover, are also able to actively conditionate distant tissues and to establish the so-called pre-metastatic niches, where a complex interplay between cellular components and tumor-secreted elements generate a fertile soil for the following secondary tumor colonization and metastasis [15].

Notably, the informational content of tumor-derived vesicles, in particular of exosomes released by the primary tumor (tumor-derived exosomes, TDEs), for paracrine and endocrine signaling, educate receiving cells in the niche and exert a key role in the establishment of a microenvironment favorable to metastasis. Therefore, the exosomes, derived both from cancer cells and from TME cellular elements, play a crucial role in all phases of the tumor process, from onset to secondary site colonization.

### Exosomes as Mediators of Intercellular Communication in Cancer

Exosomes are small extracellular vesicles with a diameter of 30–150 nm that can be produced in the endolysosomal and multivesicular body compartments by most cells and released into the extracellular environment [16]. EV complex cargo, including a wide range of biomolecules (lipids, DNA, proteins, lncRNAs, miRNAs, mRNAs, circRNAs, metabolites, and even organelles), protected from enzyme degradation by a lipid bilayer membrane, can be delivered to target cells, thus influencing their phenotype and cellular processes. The most attractive feature of exosomes is that their content reflects the pathophysiological status of the producing cells because the loading of specific molecules is finely regulated [17,18,19,20,21,22].

Several studies demonstrated the pivotal role of exosomes in cancer biology. Notably, TDEs deliver autocrine and paracrine signals that reprogram and educate the cellular components of the tumor niche, such as normal parenchymal and stromal cells, cancer-associated fibroblasts (CAFs), endothelial cells, and local immune cells, thus creating the microenvironmental conditions suitable for tumor progression [23]. Moreover, the systemic release of TDEs contributes to the establishment of the pre-metastatic niche and metastasis by reprogramming cells of secondary sites and, eventually, determining the organotropism of circulating metastatic cells and their engraftment [24].

The functional effects induced by the exosome-released cargo in recipient cells include the modulation of immunosuppression (recruitment of suppressive immune cells or inhibition of T cell activation and proliferation, impairment of the adaptive immune response), angiogenesis (activation of endothelial cells, expression/secretion of matrix metalloproteinase), local inflammation (induction of the expression/secretion of inflammatory factors from stromal and immune cells), extracellular matrix remodeling (ECM deposition by stromal cells, and exosome adhesion to ECM molecules to facilitate colonization of circulating tumor cells) [24,25].

Part of these effects can be ascribed to the EMT phenotype of the EV-producing cells in the tumoral environment. Previous studies have found that the EMT not only can occur in a subpopulation of cells within the tumor mass, triggering the generation of circulating cancer cells, but that it can also be significantly induced in other neighboring epithelial normal and transformed cells by tumor-derived exosomes, thus triggering the acquisition of a mesenchymal phenotype and metastatic properties [26], respectively. Therefore, exosomes released by EMT-induced cells can in turn confer EMT features to neighboring cells, thus producing a synergistic effect [27,28].

These overall pro-EMT signals can also be supplied by cells of the microenvironment. Among stromal cells, cancer-associated fibroblasts (CAFs) are the main cellular components of TME in most solid cancers. Several studies have described the role of CAFs in inducing EMT in tumor cells through the exosome-mediated delivery of cytokines such as TGFβ and of miRNAs, promoting cell migration, invasion, and metastasis [29,30,31,32]. In addition to stromal cells, tumor-associated macrophages (TAMs) were shown to contribute to gastric cancer cell migration through the exosome-mediated induction of EMT-related changes such as the upregulation of matrix metalloproteinases and EMT transcription factors and cytoskeleton remodeling [33]. Exosomal miRNAs from hypoxic bone marrow-derived mesenchymal stem cells (MSCs) were found to promote the invasion and metastasis of lung cancer cells by STAT3-driven EMT [34]. Cancer stem cell-derived (CSCs) exosomes were shown to accelerate the EMT-mediated tumorigenesis and metastasis in vivo of human renal clear cell carcinoma cells [35].

Therefore, cancer-related exosomes released by cancer cells, CSCs, or tumor microenvironment associated cells can contribute to a dynamic inter-cellular crosstalk, inducing and reinforcing tumor progression through the delivery of active biomolecules able to activate signaling pathways drivers of EMT.

Some of the molecules delivered by tumor-derived exosomes have been identified and include RNAs with regulative roles, such as miRNAs, lncRNAs, and circRNAs [36]. While the role of miRNAs and lncRNAs in tumor onset and progression has already been documented and largely described, the role of circRNAs in cancer-related EMT is still emerging, and its study is in full development. Recent reports revealed that cirRNAs are highly enriched and stable in exosomes compared to the producer cells [37] and closely related to cancer where they mediate multiple processes during tumor progression [38].

## 3. Circular RNAs (circRNAs)

Circular RNAs are single stranded, low copy, covalently closed-loop molecules. The lack of 5′ and 3′ ends renders them resistant to exonuclease-mediated degradation and they are therefore more stable than linear transcripts [39]. CircRNAs were discovered in eukaryotic cells about forty years ago [40] and found from archaea to mammals [41,42,43]; nevertheless, they were considered for a long time as simple splicing noise to be ignored [44]. However, recently a growing amount of literature pointed to the relevance of these RNAs in cell differentiation and physio-pathology. The evidence of different functional roles, coupled to the developmental stage/tissue specific expression and to evolutionarily conserved sequences, highlighted that the circRNA class does not include byproducts of the splicing process but molecules whose biogenesis is finely regulated. 

### 3.1. Biogenesis of circRNAs

Recent advances in high-throughput RNA-seq approaches have allowed the identification of a large number of circRNAs in non-polyadenylated transcriptomes. These molecules mainly derive from exons (exonic circRNAs or ecircRNAs); however, circRNAs that consist of both exons and introns (exonic-intronic ciRNAs or EIciRNAs) or only introns (circular intronic RNAs or ciRNAs), as well as intergenic circRNAs, have been found [45]. 

**Figure 1:**
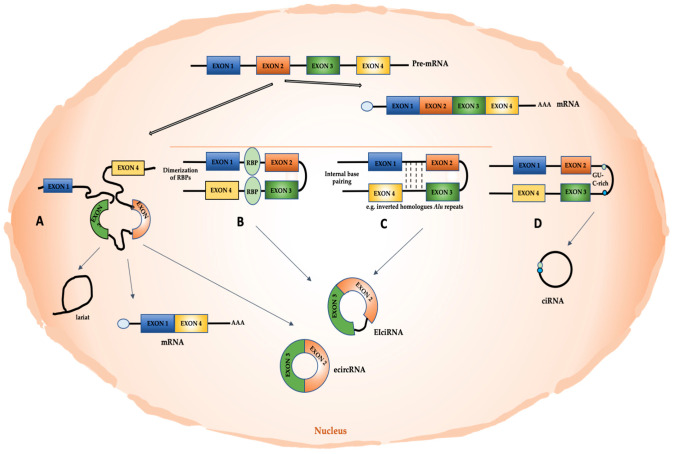
Biogenesis of circular RNAs. Pre-mRNA can be processed via canonical splicing; however, a lariat-driven RNA circularization can result in the formation of ecircRNAs, intron lariat, and linear mRNAs (**A**). Circularization in EIciRNAs or ecircRNA can depend on the bridge formed by RBP pairing (**B**) or be triggered by internal base pairing (e.g., for inverted homologous *Alu* repeats) (**C**). Lariat formation of ciRNAs can be favored by GU and C-rich elements (**D**).

Exonic circRNAs can derive from the pre-mRNA back-splicing mechanism (Figure 1), following the pairing of a downstream splice donor site with an upstream splice acceptor [46]. The back-splicing mechanism can be facilitated by intron looping mediated by internal base pairing, as may be in the presence of reverse complementary *Alu* repeats [42,47,48,49,50,51]. The interaction between distant sequences can also be favored by RNA Binding Proteins (RBPs). For instance, DHX9 acts as a negative regulator of the production of circRNAs by means of its specific binding to transcribed inverted *Alu* repeats [52]. Instead, RNA-binding proteins FUS and Quaking (QKI) induce the production of several circRNAs by binding introns bordering the back-splicing junctions [53,54]. The splicing factor musclebind (MBL/MBNL1) strongly and specifically binds to circMbl, a circular form that is generated from the second exon of its own mRNA; this interaction increases circRNA levels in dependance on the binding of MBL on intronic consensus sites flanking the exon [55]. Other RBPs are suggested to impact on back-splicing mechanisms, including the heterogeneous nuclear ribonucleoprotein L (HNRNPL) [56]. Interestingly, Dudekula et al. used an innovative web tool (CircInteractome, for circRNA interactome) to map the RBP-binding sites in the flanking sequences upstream and downstream of mature circRNAs. By specifically using circ_0000020, they provided evidence of multiple binding sites for HuR, FMRP, and EIF4A3 [57]. Notably, a recent work by Minzhe et al. proposed a pipeline, called Clirc, to profile circRNAs in CLIP-Seq datasets and to analyze circRNA-RBPs interactions [58]. 

A further mechanism of circRNA biogenesis, discovered in *S. pombe*, implies the formation of a lariat exon-containing precursor, whose internal splicing causes the removal of the intronic sequence and the circularization of the exon (Figure 1) [59]. This mechanism can explain circRNA biogenesis when sequences facilitating a direct back-splicing are lacking.

In ciRNA biogenesis, exons can also be circularized, retaining introns between them, thus originating EIciRNAs [60]. Furthermore, ciRNA biosynthesis may depend on a failure in debranching and requires a consensus motif containing a 7nt GU-rich element near the 5′ splice site and an 11nt C-rich element near the branchpoint site [61].

Interestingly, by applying a new chiastic clipping signal-based algorithm (CIRI) to ENCODE RNA-seq data, Gao et al. also identified intergenic circRNAs in human transcriptome [62].

From all that has been said so far, it is clear that the formation of circRNAs is a finely regulated process; therefore, how much and which circRNAs are produced by the same precursor RNA may depend on the cell type and on the particular conditions in which cells and tissue are found.

Furthermore, different circRNAs produced starting from the processing of the same precursor RNA can exert different and even opposite effects on the same cellular process. This has to be taken into consideration when mentioning, with the same name, circRNAs that may only share their origin.

### 3.2. Mechanisms of Action of circRNAs

CircRNAs have been described as regulating gene expression through different molecular mechanisms.

CircRNAs can exert both transcriptional and translational control of the expression of their parental genes. EIciRNAs (such as circEIF3J and circPAIP2) localize in the nucleus, where they can form a complex with U1 snRNP and Pol II at promoter regions of parental genes and regulate their transcription in *cis*. Notably, this function requires the interaction between the circRNA and U1 snRNA [60]. Similarly, ciRNAs can positively modulate Pol II elongation activity at their own genes [61].

CircRNAs in some cases function as negative regulators of their own linear mRNAs. Some of them, in fact, contain the translation start codon thus precluding that the protein coding transcripts from which they derived can be translated [42,63]. Furthermore, circPABPN1 (has_circ_0031288) arises from the PABPN1 mRNA and can bind to HuR, thus interfering with the capacity of this RBP to interact with PABPN1 mRNA. This suppresses its translation, modulated positively by HuR [64]. Notably, the circular transcript of the YAP gene, circYAP, forms a complex with YAP mRNA and the translation initiation site-associated proteins eIF4G and PABP. In this manner, it blocks the interaction of PABP on the 3′ tail with eIF4G on the 5′-cap, thus suppressing YAP mRNA translation initiation [65]. Another remarkable mechanism of post-transcriptional downregulation is used by circMbl. The MBL protein is a general splicing factor, playing a crucial role both in canonical splicing and in the circularization of its own mRNA, depending on its level. When the protein level is high, circMbl production increases, while the production of its own mRNA decreases. Moreover, MBL protein is massively recruited by intronic binding sites retained in the circRNA. This adds a further level of negative control of circMbl on its own MBL protein coding linear transcript [55].

Notably, circRNAs can act as decoys for proteins involved in gene regulation. CircVPS13C functions as a key regulator of pituitary adenoma growth by competitively interacting with a ribosome-binding protein of the endoplasmic reticulum membrane, thus decreasing the stability of the mRNA coding for the antiproliferative protein [66]. A further example of how circRNAs can impact gene regulation by modulating the stability of the transcript comes from circRasGEF1B. It acts as a regulator of lipopolysaccharide (LPS) response in macrophage cells by regulating the stability of ICAM-1 mRNA [67]. Otherwise, circPan3 controls self-renewal capacity of multipotent intestinal stem cells (ISCs) by protecting the mRNA of an IL-13 receptor subunit from KSRP-mediated degradation [68]. 

A large body of evidence has proven that circRNAs can act as miRNA sponges, thus competing with target mRNAs for their binding and interfering with the miRNA-mediated post-transcriptional regulation of gene expression. Hansen et al. showed that ciRS-7 (circular RNA sponge for miR-7, also known as CDR1as, i.e., cerebellar degeneration related protein 1 antisense circRNA), identified in human and mouse brains, contains more than 70 target sites for miR-7. CiRS-7 overexpression strongly suppresses miR-7 activity in neocortical and hippocampal neurons, resulting in an increased level of its targets [69,70]. The same model of action was demonstrated for several other circRNAs, for example: circ-SRY (for sex-determining region Y) sponges miR-138 in mice testes [69]; circ-ITCH, derived from several exons of itchy E3 ubiquitin protein ligase (ITCH), sponges miR-7, miR-17 and miR-214, in esophageal squamous cell carcinoma [71], and miR-214 also in osteogenic differentiation [72].

Moreover, other circRNA functions include the capacity of forming complexes with specific proteins by acting as scaffolds. Protein binding may result in their sequestration or in the modulation of their compartmentalization. For example, Du et al. reported that the circ-Foxo3 negatively controls the cell cycle progression by binding CDK2 and p21 and impairing their function [73]. The same circRNA interacts in the cytoplasm with the anti-senescent protein ID-1 and the transcription factor E2F1, as well as the anti-stress proteins FAK and HIF1α, impairing their anti-senescent and anti-stress roles [74]. CircACC1, instead, exerts a critical role in controlling cellular response to metabolic stress by binding to regulatory subunits of the AMPK complex and regulating its assembly [75].

Furthermore, recent evidence indicates that circRNAs can be translatable, even if they lack the 5′ cap and the 3′ poly(A) tail. Specifically, Zhang et al. demonstrated that the circular RNA transcribed by the SNF2 histone linker PHD RING helicase (SHPRH) gene can be translated into a 17 kDa SHPRH-146aa protein that protects the full-length SHPRH from degradation by the ubiquitin proteasome, leading to inhibition of cell proliferation and tumorigenicity in glioblastoma [76]. Moreover, Legnini et al. reported that circ-ZNF609 can be translated in a cap-independent manner and functions in myogenesis by controlling myoblasts proliferation [77].

**Figure 2:**
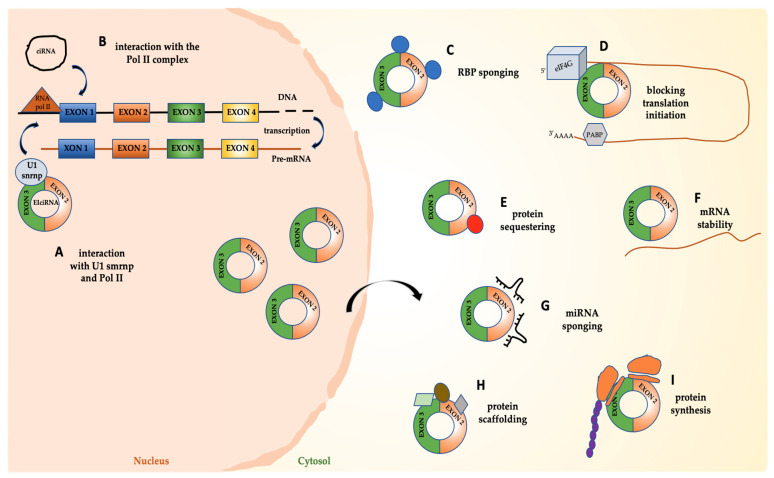
Functional mechanisms of circRNAs. EIciNAs can form a complex with U1 small nuclear ribonucleoprotein (U1 snRNP) and bind RNA PolII to enhance transcription (**A**). CiRNAs can interact with RNA PolII to positively modulate transcription (**B**). CircRNAs can act as sponges of RBPs, thus impacting their pleiotropic functions (**C**); they can control translation initiation (**D**), protein sequestering, localization or function (**E**). Moreover, circRNAs can affect mRNA stability (**F**); sponge miRNAs, thus interfering with the post-transcriptional regulation of their target mRNAs (**G**), or act as scaffold for proteins (**H**). Some circRNAs can also be translated (**I**).

Mechanisms of function of circRNAs are depicted in Figure 2.

## 4. Functional Role of circRNAs in EMT and Tumor Progression

Increasing evidence supports a regulatory role of circRNAs in cancer-associated EMT and are mainly referred to studies on human tissues or cellular models, even if circRNAs show a high degree of conservation across species and their mechanisms of action have been studied in different organisms, including the mouse [43].

Firstly, the expression of circRNAs is modulated during the transition, and EMT transcription factors (EMT-TFs) can be directly involved in controlling their expression. For example, in hepatocellular carcinoma, Twist positively controls circCul2 (circ10720) expression to increase vimentin levels. This circRNA, indeed, sponges miR-1246, miR-578, and miR-490-5p, targeting this mesenchymal marker [78].

Secondly, a growing amount of evidence points to the functional role of circRNAs as competing endogenous RNAs in controlling the levels of both EMT-TFs and signaling molecules involved in the induction of the transition.

In melanoma, circRNA_0084043 promotes cell proliferation, migration, and invasion by sponging miR-153-3p, then determining Snail upregulation [79]. In cervical cancer, circRNA-000284 induces tumor cell proliferation and invasion by sponging miR-506, which targets Snail-2 [80]. Similarly, circPRMT5 binds to miR-30c in cells of urothelial carcinoma of the bladder, thus modulating the levels of its target Snail [6]. Another circRNA involved in the metastatic EMT of bladder cancer is circ-HIPK3, produced from exon 2 of the homeodomain-interacting protein kinase 3 (HIPK3) gene, also known as bladder cancer-related circular RNA-2 (BCRC-2). It sponges miR-558 in bladder cancer cells [81]; interestingly, miR-558 suppresses heparinase, whose expression has been closely related to EMT [82]. Moreover, circHIPK3 serves as a sponge for multiple other miRNAs in human cells, exhibiting a pleiotropic activity [83]. In papillary thyroid cancer, circNUP214 sponges miR-145, causing the upregulation of ZEB2 and promoting cell proliferation, migration, and invasion (PTC) [84]. In non-small cell (NSC) lung cancer cells, circAGFG1 enhances proliferation, invasion, and migration by acting as competing endogenous RNA of miR-203 and positively regulating its target, ZNF281 [85]. Moreover, in breast cancer, circANKS1B (hsa_circ_0007294) sponges miR-148a-3p and miR-152-3p, leading to the increase of the expression of the transcription factor USF1, in turn activating TGF-β1/Smad signaling [86]. In breast cancer progression is also involved circ-ROBO1 (has_circ_0124696), which promotes breast cancer-derived liver metastasis by sponging miR-217-5p [87], known to target Zeb1 [88]. Furthermore, in oral squamous cell carcinoma cells, circUHRF1 (hsa_circ_0002185) exhibits a sponge activity for miR-526b-5p, thereby regulating c-Myc and promoting TGF-β1 transcription [89]. Myc is also a target for miR-449c-5p and circNOTCH1, upregulated in gastric cancer and promotes tumor growth and metastasis by interfering with miR-449c-5p/MYC/NOTCH1 axis [90].

However, circRNAs can also act as sponges to negatively regulate the EMT process. This is the case with circAKT3 (hsa_circ_0017252), repressed in clear cell renal cell carcinoma metastasis, which binds to miR-296-3p and interferes with the post-transcriptional down-regulation of E-cadherin mediated by this miRNA [91]. Moreover, the circAMOTL1L (hsa_circRNA_000350) down-regulation is required in EMT of prostate cancer cells, in the light of the sponge activity of this circRNA on miR-193a-5p, known to target a subset of cadherin superfamily members [92]. Similarly, in lung cancer cells, hsa_circ_0007059 overexpression causes up-regulation of E-cadherin and inhibition of Vimentin, Twist, and Zeb1. This circRNA impairs miR-378 activity, thus negatively regulating the EMT process via inactivation of the Wnt/β-catenin and ERK1/2 pathways [93]. Wnt/βcatenin signaling can also be interfered in triple-negative breast cancer by circ-ITCH. It was found significantly downregulated in tumor cells, and its restoration was shown to inhibit proliferation, invasion, and metastasis by sponging miR-214 and miR-17 [94]. Circ-ITCH also inhibits bladder cancer progression by sponging miRNAs targeting PTEN [95], known to suppress EMT in cancer stem cells [96]. Circ0026344 restrains EMT by suppressing miR-183-dependent Wnt/β-catenin signaling in colon cancer [97]. In small cell lung cancer cells, cESRP1 binds to miR-93-5p, upregulating its target Smad7, a negative feedback regulator of the TGF-β-mediated signaling [98]. Another oncosuppressor is circPTPRA, which, in non-small lung cancer cells, suppresses EMT in vitro and in vivo by sequestering miR-96-5p and upregulating RASSF8 [99]. An inhibitory effect on proliferation and metastasis of human cancers has been attributed to circPTK2. In non-small cell lung cancer tissues, TGF-β down-regulates circPTK2 (hsa_circ_0008305) that functions as a sponge of the pro-EMT regulators miR-429 and miR-200b-3p. Conversely, circPTK2 overexpression significantly decreases the expression of Snail and limits the invasiveness of tumor cells [100]. An anti-oncogenic effect of circPTK2 has also been observed in glioblastoma [101] and in gastric cancer where it sponges miR-196a-3p, a down-regulator of the oncosuppressor gene AATK [102]. Interestingly, from the same PTK2 pre-mRNA are derived another two circRNAs, circPTK2 (hsa_circ_0005273) and circPTK2 (hsa_circ_0003221), containing different spliced sequences and exerting oncogenic functions in colorectal [103] and bladder cancer [104] as well as laryngeal squamous cell carcinoma [105]. CircST6GALNAC6 (hsa_circ_0088708) is downregulated in bladder cancer tissues and cells. This circRNA inhibits the EMT in vitro and the metastasis process in vivo. Mechanistically, Tan et al. demonstrated that circST6GALNAC6 serves as a sponge that directly binds miR-200a-3p [106], the main regulator of EMT [107]. CircEHMT1, whose levels are strongly decreased in breast cancer tissues, inhibits migration and invasion of tumor cells targeting miR-1233-3p that, in turn, control MMP2 by means of the transcription factor KLF4 [108].

Beside sponge activity, the functional role of circRNAs can include the binding with specific proteins to modulate the activation of molecular pathways involved in EMT induction. For example, circPTK2 (hsa_circ_0005273) can promote EMT of colon cancer cells by binding to vimentin on sites Ser38, Ser55, and Ser82 [103]. Moreover, Bi et al. proposed that circRNA_102171 promotes proliferation, migration and invasion of papillary thyroid cancer cells by interacting with CTNNBIP1. This binding interferes with the association of this protein with the β-catenin/TCF complex and causes the activation of the Wnt/β-catenin pathway [109].

Furthermore, Pan et al. demonstrated that circFNDC3B negatively controls the EMT in colon cancer progression via encoding a protein, named circFNDC3B-218aa, able to inhibit Snail expression [110].

Finally, in breast cancer, circYAP is negatively regulated; this circRNA, indeed, suppresses translation initiation of YAP protein [65], a key regulator of the EMT [111].

CircRNAs involved in EMT are listed in Table 1.

## 5. Exosomal circRNAs in Cell-Tumor Microenvironment Crosstalk

EV-associated circRNAs exert a key role in the modulation of EMT features as mediators of communication between primary tumor cells and TME. Accordingly, circRNAs in biological fluids are valuable as non-invasive biomarkers for diagnosis of different cancer stages, independently of which EV species (often not investigated) delivers them [7]. However, growing evidence points to the exosomes as vehicles of circRNAs in the cell-tumor microenvironment crosstalk.

At first, tumor cells with high metastatic potential can donate to recipient epithelial cells exosomes whose specific cargo of circRNAs confers them EMT features and pro-metastatic properties. This is accomplished through the induction of signaling pathways triggering the transdifferentiation process or the positive modulation of mesenchymal markers. In vitro studies demonstrated that exosomes from high metastatic LM3 hepatoma cells increase the migration and invasion potential of non-metastatic (HepG2) and low metastatic (97L) cells, and these effects are blocked by circPTGR1 silencing. Mechanistically, the sponge activity of circPTGR1 on the tumor suppressor miR-449a results in the positive regulation of its target MET, in turn modulating the phenotypic change. Notably, in HCC the prognosis of patients with low exosomal circPTGR1 levels in serum is better than that of patients with high expression of this circRNA [113,127]. Low overall survival of HCC patients also correlates with high levels in the tumor of circMMP2 and low levels of miR-136-5p. In vitro circMMP2, delivered by exosomes from highly invasive cells, confers EMT properties to normal hepatocytes and nonmetastatic HepG2 cells; conversely, circMMP2 silencing in HCC cells weakens their ability to metastasize. Molecularly, it has been shown that circMMP2 causes the upregulation of MMP2 expression via sponging miR-136-5p [114]. CircRNA100338 is a further exosomal circRNA produced by highly invasive HCC cells that positively regulates the metastatic capacity of recipient hepatocytes and hepatoma cells, upregulating MMP9 expression. Accordingly, high levels of this circRNA in serum of HCC patients have been proposed as a risk indicator of metastasis and poor survival [115]. Interestingly, the exosomal circRNA-mediated upregulation of MMP2 is also involved in the process of ovarian cancer cell metastasization. CircPUM1, indeed able to upregulate MMP2 by sponging of miRNA-6753-5p and miR-615-5p, is expressed by cancer cells and transferred to peritoneum. Here, overexpressed MMP2 participates in the process of the mesothelium-to-mesenchymal transition, favoring cancer dissemination [116].

In gastric cancer, exosome-embedded circ133 is delivered from hypoxic cells to normoxic cells and promotes cell migration by acting on the miR-133a/GEF-H1/RhoA axis. Furthermore, this circRNA is enriched in the plasma exosomes of patients, and its levels increase with tumor progression [117]. Metastasis and poor prognosis in gastric cancer also correlate with circNHSL1 upregulation. Furthermore, in vitro studies have demonstrated that this circRNA has an oncogenic role by sponging miR-1306-3p and relieving the repression of its target SIX1. This transcriptional factor, in turn, induces the expression of the mesenchymal marker vimentin [118]. Interestingly, SIX1 promotes EMT of mammary carcinoma cells by increasing TGFβ signaling [128] and regulates the transdifferentiation of colorectal cancer cells by post-transcriptional activation of the EMT-TF ZEB1 [129]. Moreover, circ-0000284 expression levels are high in cholangiocarcinoma cell lines and tumor tissues. This circRNA is secreted in exosomes and delivered to surrounding normal cells to promote their migration and proliferation by acting as competitive endogenous RNA [119].

In laryngeal squamous cell carcinoma progression, the levels of circRASSF2 are significantly elevated in exosomes from plasma and tumor tissues. As reported by Tian et al., this circRNA modulates the miR-302b-3p/insulin-like growth factor 1 receptor (IGF-1R) axis, thus promoting proliferation, migration, and invasion of tumor cells [120]. Notably, miR-302 is known to induce mesenchymal-epithelial transition during reprogramming of fibroblasts and to block TGFβ-induced EMT of epithelial cells [130].

Additionally, untransformed epithelial cells can communicate via EV-associated circRNAs to in situ tumor cells to counteract their malignant behavior and promote cell apoptosis or the arrest of the cell cycle. For example, exosome-embedded circ0051443 is produced by normal hepatocytes and delivered to hepatocarcinoma cells in which it regulates BAK1 expression through competitive binding to miR-331-3p, thus blocking tumor progression. Accordingly, circ-0051443 levels are significantly lower in the plasma exosomes and tissues from HCC patients than healthy controls [121]. Of note, miR-331-3p acts as a negative regulator of EMT, migration and metastasis also in non-small-cell lung cancer, by targeting ErbB2 and VAV2 (that control Rac1, PAK1, and β-catenin) [131].

CircRNA-containing exosomes are also key regulators of the crosstalk between tumor cells and non-transformed stromal host cells, including endothelial cells, fibroblasts, and immune cells. EV-delivered circRNAs can regulate angiogenesis induction, invasion, and metastasis activation as well as immune surveillance evasion.

For example, HCC cells secrete circUHRF1 via exosomes, and high levels of this circRNA in HCC patient plasma not only correlates to poor clinical prognosis but also to NK cell dysfunction. CircUHRF1 acts as a sponge for miR-449c-5p, determining in NK cells the upregulation of its target TIM-3 that, in turn, inhibits IFN-γ and TNF-α secretion and the antitumoral immunity [112]. It can be supposed that miR-449c-5p regulation in primary tumor cells can also affect their ability to metastasize, in analogy to that reported in gastric cancer, where miR-449c-5p function is inhibited by circNOTCH1 to modulate Myc and Notch1 [90]. Interestingly, Notch signaling positively regulates the EMT, invasion, and growth of breast cancer cells by inducing Slug expression [132]. Moreover, circRNA100338, secreted in exosomes by HCC highly invasive cells, is internalized by HUVEC cells and binds to several RBPs, affecting cell proliferation, angiogenesis, and permeability [115]. A role for circASAP1 (hsa_circ_0085616) has been recently reported in HCC where it upregulates Mitogen-Activated Protein Kinase 1 expression by sponging miR-326 and miR-532-5p, thus inducing high metastatic properties in cancer cells and regulating TAM infiltration [122].

Furthermore, Xu et al. reported that circ-CCAC1 from cholangiocarcinoma-derived EVs transferred to endothelial monolayer cells induces the disruption of endothelial barrier integrity and promotes angiogenesis. Circ-CCAC1 acts by sequestering EZH2 in the cytoplasm, thereby preventing its nuclear translocation. This determines the positive regulation of SH3GL2, a negative regulator of ZO-1 and occludin. Furthermore, circ-CCAC1 regulates the miR-514a-5p/YY1 axis by means of a competitive endogenous RNA mechanism, thus controlling the proliferation and invasiveness of producing cholangiocarcinoma cells [123].

Moreover, EVs can be produced by cells in TME and internalized by tumor cells impacting their phenotype. For example, in vitro and in vivo experiments have shown that circ-DB secreted in EVs from adipocytes promotes the growth of HCC cells by inhibiting miR-34a and activating deubiquitination-related USP7 [124]. Notably, miR-34a is a key EMT regulator, inhibiting several EMT-TFs [133].

Tumor-associated macrophages (TAM) are further regulators of tumor progression through EV-associated circRNAs. In cholangiocarcinoma, proliferation, migration, and invasion are modulated by circ_0020256 conveyed by TAM-secreted exosomes. This circRNA acts via miR-432-5p sponging to determine the rescue of its-mediated repression of the transcription factor E2F3. In fact, crc_0020256 overexpression causes the downregulation of E-cadherin expression and the upregulation of N-Cadherin, and these effects can be reversed by the miR-432-5p mimics [125].

In the molecular interplay between cancer cells and the surrounding microenvironment, a role is played by CAFs, able to secrete several pro-tumoral molecules. Little is known at present about CAF-derived exosomal circRNAs. The CAF-derived exosomal circSL7A6 has been proposed to play a role in EMT by sequestering miRNA21 and the anti-EMT miRNA-200 members in colon cancer [126]. Interestingly, a role for circHIF1A, found in exosomal cargo of CAFs in breast cancer, has been recently reported to modulate the expression of CD44 and ultimately induce stemness properties to cancer cells [134].

**Figure 3:**
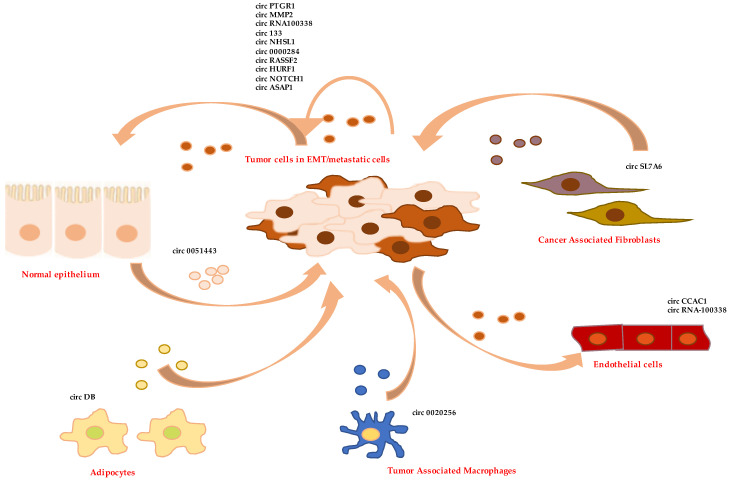
Exosome-mediated cell-to-cell communication in TME. Exosomes-associated pro- and anti-EMT circRNAs in the communication between tumor cells and surrounding microenvironmental elements, including normal epithelial cells, tumor-associated macrophages, endothelial cells, cancer-associated fibroblasts, and adipocytes.

The EV-associated circRNAs involved in EMT are listed in Table 1, and their involvement in mediating cell-to-cell communication are depicted in Figure 3.

## 6. Conclusions and Perspectives

EMT has a key role in tumor progression and metastasis, and this makes the molecules and signaling pathways involved in this process attractive targets for therapeutic approaches. However, this process is not of the “all or nothing” type, and the complex interplay between cells in EMT and tumor niche demands a great attention in developing possible strategies.

It is a matter of fact that the recent identification of distinct circRNAs, enriched in exosomes from cells undergoing EMT, as key mediators of the crosstalk between cells of the TME, strongly encourages the use of these molecules as novel biomarkers of invasive tumors. Moreover, while more efforts are needed to clarify the role of single molecules in invasive tumor networks, recent findings, as described above, already suggest that specific circRNAs could be conceivably addressed to achieve targeted therapeutic intervention, as previously proposed for other RNAs (i.e., miRNAs and lncRNAs) with a better documented role in cancer development and progression. A therapeutic approach targeting specific circRNAs enriched in exosomes could be able, indeed, to limit the cell-to cell communication that fosters tumor outcome. Further studies are required to clarify the contribution in EMT of circRNAs delivered by microvesicles or if they can also be delivered by exomeres in TME [135].

As discussed above, circRNAs can exert an oncogenic or a suppressive role in tumor progression. In the first case, siRNAs targeting the back-splice junction sequences may provide a simple strategy to achieve potential beneficial outcomes. Conversely, circRNAs with anti-EMT roles could be overexpressed or delivered by exosomes. Being circular molecules, they exhibit a great stability that represents an advantage with respect to linear RNAs and would allow a long-term expression at lower dosage. Interestingly, overexpression vectors, including lentivirals and adeno-associated viral vectors, have been successfully used in vitro and in vivo for expression and translation of circRNAs [136,137,138,139]. However, the more promising strategies of silencing or overexpression are based on possible EV-based therapeutic approaches, that should imply the engineering of EV-producing cells where the vesicles are enriched with the nucleic acids of interest.

This could be a limiting step, taking in account that the cellular mechanisms controlling the sorting of specific molecules are still poorly known. However, the use of nanoparticles or synthetic exosomes as carriers can be conceived for better results [140]. Interestingly, Kojima et al. reported a set of EXOsomal transfer into cell (EXOtic) devices for the efficient and customizable production of exosomes by mammalian cells and demonstrated the delivery of mRNA cargo by engineered producing cells implanted in mice [139]. Furthermore, nanoparticles have been successfully used for the delivery in mice of constructs overexpressing circEHMT1, an anti-EMT circRNA, as a therapeutic approach for breast cancer [108].

## Figures and Tables

**Table 1:** CircRNAs with a role in EMT and tumor progression.

circRNA	Putative Function	Type of Cancer/EMT Cells	Effect on EMT/Metastasis	Localization	Reference
circPRMT5	miRNA sponge	bladder carcinoma	pro	not investigated	[6]
circYAP	translation initiation	breast cancer	anti	not investigated	[65]
circCul2	miRNA sponge	HCC	pro	not investigated	[78]
circ_0084043	miRNA sponge	melanoma	pro	not investigated	[79]
circRNA-000284	miRNA sponge	cervical cancer	pro	not investigated	[80]
circHIPK3	miRNA sponge	different cancers	pro	not investigated	[81,83]
circNUP214	miRNA sponge	thyroid cancer	pro	not investigated	[84]
circAGFG1	miRNA sponge	NSC lung cancer	pro	not investigated	[85]
circANKS1B	miRNA sponge	breast cancer	pro	not investigated	[86]
circROBO1	miRNA sponge	breast cancer	pro	not investigated	[87]
circUHRF1	miRNA sponge	oral squamous carcinoma/HCC	pro	not investigated/ exosome	[89,112]
circNOTCH1	miRNA sponge	gastric cancer	pro	exosome	[90]
circAKT3	miRNA sponge	clear renal cancer	anti	not investigated	[91]
circAMOTL1L	miRNA sponge	prostate cancer	anti	not investigated	[92]
hsa_circ_0007059	miRNA sponge	lung cancer	anti	not investigated	[93]
circITCH	miRNA sponge	TNBC/bladder carcinoma	anti	not investigated	[94,95]
circ_0026344	miRNA sponge	colon cancer	anti	not investigated	[97]
circESRP1	miRNA sponge	small cell lung cancer	anti	not investigated	[98]
circPTPRA	miRNA sponge	NSC lung cancer	anti	not investigated	[99]
circPTK2 (hsa_circ_0008305)	miRNA sponge	different cancers	anti	not investigated	[100,101,102]
circPTK2 (hsa_circ_0005273)	protein binding	colon cancer	pro	not investigated	[103]
circPTK2 (hsa_circ_0003221)	miRNA sponge	different cancers	pro	not investigated	[104,105]
circST6GALNAC6 (hsa_circ_0088708)	miRNA sponge	bladder cancer	anti	not investigated	[106]
circEHMT1	miRNA sponge	breast cancer	anti	not investigated	[108]
circRNA_102171	decoy for protein	papillary thyroid cancer	pro	not investigated	[109]
circFNDC3B	translated in protein	colon cancer	anti	not investigated	[110]
circPTGR1	miRNA sponge	HCC	pro	exosome	[113]
circMMP2	miRNA sponge	HCC	pro	exosome	[114]
circRNA_100338	not investigated	HCC	pro	exosome	[115]
circPUM1	miRNA sponge	ovarian cancer	pro	exosome	[116]
circ133	miRNA sponge	gastric cancer	pro	exosome	[117]
circNHSL1	miRNA sponge	gastric cancer	pro	exosome	[118]
circ-0000284	not investigated	cholangiocarcinoma	pro	exosome	[119]
circRASSF2	miRNA sponge	laryngeal squamous cell carcinoma	pro	exosome	[120]
hsa_circ_0051443	miRNA sponge	HCC	anti	hepatocyte-secreted exosome	[121]
circASAP1	miRNA sponge	HCC	pro	exosome	[122]
circCCAC1	protein decoy and miRNA sponge	cholangiocarcinoma	pro	exosome	[123]
circ-DB	miRNA sponge	HCC	pro	adipocyte-secreted exosomes	[124]
circ_0020256	miRNA sponge	cholangiocarcinoma	pro	TAM-secreted exosomes	[125]
circSL7A6	miRNA sponge	colon cancer	pro	CAF-secreted exosomes	[126]

## Data Availability

Not applicable.
